# Fenestrierte und verzweigte endovaskuläre Aortenprothesen: Ein Update

**DOI:** 10.1007/s00117-022-01019-1

**Published:** 2022-06-20

**Authors:** Theresa-Marie Dachs, Sven Rudolf Hauck, Maximilian Kern, Catharina Klausenitz, Martin A. Funovics

**Affiliations:** 1 Abteilung für Kardiovaskuläre und Interventionelle Radiologie, Universitätsklinik für Radiologie und Nuklearmedizin, Medizinische Universität Wien, Währinger Gürtel 18–20, 1090 Wien, Österreich; 2 Institut für Radiologie, Klinik Floridsdorf, Wien, Österreich

**Keywords:** Aortenaneurysma, Endovaskuläre Aortenreparatur, Verzweigte Endoprothese, Fenestrierte Endoprothese, Verbindungsstentgraft, Komplexe Aortenreparatur, Aortic aneurysm, Endovascular aortir reapir, Branched endoprosthesis, Fenestrated endoprosthesis, Connecting stentgraft, Complex aortic repair

## Abstract

**Hintergrund:**

Komplexe abdominelle aortale Pathologien, welche die Abgänge der Viszeralarterien miterfassen und bei denen kein adäquater proximaler Hals gegeben ist, können heute mittels fortgeschrittener FEVAR/BEVAR-Technik („fenestrated/branched endovascular aneurysm repair“) mit ähnlicher Sicherheit und vergleichbaren Erfolgsraten behandelt werden wie infrarenale Pathologien mit konventionellem EVAR.

**Methodische Innovationen und Probleme:**

Zur Versorgung der Viszeralarterien können Fenestrierungen (bei Abgang der Viszeralarterie aus der nichtdilatierten Aorta) oder Verzweigungen (bei Abgang aus der dilatierten Aorta) verwendet werden. Beide Arten von Öffnungen werden mit Verbindungsstentgrafts (VSG) zu den Viszeralarterien abgedichtet. Mehrere Hersteller bieten fenestrierte oder verzweigte Endoprothesen an, wobei diese nur in Einzelfällen CE-zertifiziert und überwiegend in Europa als individuelle Sonderanfertigungen patientenbezogen erhältlich sind. Dies setzt eine entsprechende Lieferzeit voraus, was die Behandlung akuter Patienten mit solchen Prothesen unmöglich macht. Es liegen allerdings zwei Produkte von vierfach verzweigten Endoprothesen vor, die einen größeren Bereich der anatomischen Gegebenheiten bei thorakoabdominellen Aneurysmen auch im Akutfall abdecken und behandelbar machen. Sämtliche FEVAR- und BEVAR-Hauptkörper benötigen VSG, die durchgehend von Fremdherstellern stammen und von denen gegenwärtig noch kein einziges Produkt für diese Anwendung zertifiziert ist.

**Empfehlungen:**

Da Probleme an Verbindungsstentgrafts eine wesentliche Ursache für Reinterventionen sind, sollte in der Nachsorge Knickbildungen und Brüchen an diesen Verbindungsstents besonderes Augenmerk geschenkt und von der Verwendung einschichtiger Designs beim BEVAR abgesehen werden.

## Abstract (en)

**Background:**

Today, complex abdominal aortic pathologies involving the visceral arteries and without an adequate proximal neck can be treated using fenestrated/branched endovascular aortic repair (FEVAR/BEVAR) with similar safety and success rates as infrarenal aortic pathologies treated with tubular EVAR.

**Methodical innovations and challenges:**

Fenestrations (if the vessel originates from a nondilated aorta) or branches (if the vessel originates from a dilated aorta) may be used for the visceral arteries. Both types of openings are sealed via bridging stent grafts that connect to the target vessel. Multiple manufacturers offer fenestrated or branched endoprostheses, with only a few being CE certified and the majority in Europe being patient-specific custom-made devices. Therefore, they require a certain delivery time which precludes acute patients from such treatment. However, two stent grafts with four branches for thoracoabdominal aneurysms are available off the shelf and are anatomically suitable for the majority of patients, thus, allowing for acute treatment. All FEVAR and BEVAR main bodies require bridging stent grafts, all of which are used off-label.

**Recommendations:**

As bridging stent grafts are one of the main reasons for reinterventions, one should be aware of fractures and kinking of the bridging stent grafts during follow-up and should refrain from using single-layered bridging stent grafts in BEVAR.

Die endovaskuläre Versorgung („endovascular aneurysm repair“, EVAR) von Aortenpathologien mit tubulären und Bifurkationsprothesen gilt heute als eine etablierte Behandlungsform und weist im Fall eines infrarenalen Aortenaneurysmas im Vergleich zur offenen Operation ähnliche Langzeitergebnisse und postoperativ eine bessere Morbidität und Mortalität auf [22, 24]. Die wesentliche anatomische Limitation für die Anwendbarkeit einer konventionellen endovaskulären Aortenprothese ist die Notwendigkeit einer adäquaten infrarenalen Landezone, die idealerweise gerade, nichtdilatiert und frei von größeren Thromben und Verkalkungen ist, bei einer Länge von zumindest 10–15 mm [14, 26, 27]. Anwendungen einer konventionellen Aortenendoprothese in weniger geeigneten Landezonen sind stets mit einer mehr oder weniger deutlichen Einschränkung der Erfolgsrate und der Stabilität verbunden, obschon auch hier Studien laufen, welche die Grenzen der Anwendbarkeit ausloten [1, 18].

Alternativ wurde durch die Entwicklung von fenestrierten und verzweigten aortalen Endoprothesen die Möglichkeit geschaffen, die Landezone beliebig weit nach kranial bis in die thorakale Aorta zu verlängern bei gleichzeitiger Versorgung der Viszeralarterien (Truncus coeliacus, Arteria mesenterica superior, Arteriae renales; [17]). Die fenestrierte („fenestrated“) und die verzweigte („branched“) endovaskuläre aortale Reparatur (FEVAR, BEVAR) hat speziell in Europa weite Akzeptanz gefunden und wird in den rezenten gefäßchirurgischen europäischen Richtlinien, unabhängig vom klinischen Risiko, bei geeigneter Anatomie als Behandlungsmethode der ersten Wahl empfohlen [30]. Im Vergleich zur offenen chirurgischen Versorgung wurden geringere Mortalitäts- und Komplikationsraten beschrieben [7, 8, 16, 21, 25, 28].

Die zunehmende Verbreitung von FEVAR/BEVAR ging einher mit einer kontinuierlichen Verbesserung der operativen Methodik und des verfügbaren Materials. Heute bieten in Europa – teils CE-zertifiziert, teils als patientenbezogene Sonderanfertigung – mehrere Hersteller fenestrierte und verzweigte Endoprothesen mit inneren oder äußeren Abzweigungen oder eine Kombination beider Varianten an. Alle diese aortalen Endoprothesen benötigen derzeit noch Verbindungsstentgrafts (VSG) für die Viszeralarterien, die durchweg von Fremdherstellern geliefert werden und derzeit noch nicht für eine derartige Anwendung CE-zertifiziert sind. Im Folgenden werden die aktuellen Produkte der verfügbaren Hersteller beschrieben, ihre Anwendung erläutert und auf die derzeit laufende Studiensituation eingegangen. Die Eigenschaften der Verbindungsstentgrafts werden in einem gesonderten Kapitel diskutiert.

## Fenestrierte Endoprothesen

Grundsätzlich kommen Fenestrierungen in aortalen Prothesen dort zur Anwendung, wo der Aortendurchmesser am Abgang der Viszeralarterie den maximalen Stentgraftdurchmesser nicht übersteigt (in der Regel 33–36 mm) und die Fenestrierung folglich mit Wandkontakt direkt über das Ostium der Viszeralarterie gelegt werden kann. Die Abdichtung erfolgt beim Einsetzen des Verbindungsstentgrafts zwischen diesem und dem verstärkten ringförmigen Rand der Fenestrierung, der einen Durchmesser von ca. 1 mm aufweist, im Rahmen einer forcierten Nachdilatation des Verbindungsstentgrafts am Durchtritt [29].

### Zenith Fenestrated AAA Endovascular Graft

**Figure 1:**
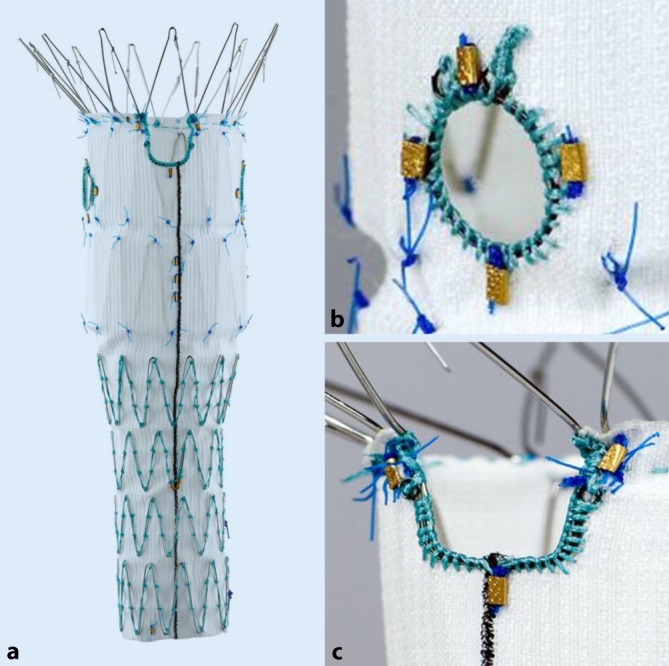

Der Zenith Fenestrated AAA Endovascular Graft (ZFEN, Cook Medical, Bloomington, IN, USA) ist eine tubuläre Endoprothese, die bis zu 5 Fenestrierungen und/oder eine proximale Einkerbung mit 10 oder 20 mm Breite aufweisen kann. In Europa sind diese Prothesen mit bis zu 2 Fenestrierungen CE-zertifiziert, was darüber hinausgeht, ist als patientenspezifische Sonderanfertigung erhältlich. Die Fenestrierungen weisen zur Verbesserung der Abdichtung einen verstärkten Ring auf und sind mit 8 mm (üblicherweise für die Viszeralarterien) oder mit 8 × 6 mm (für die Nierenarterien) erhältlich. Anstelle einer Fenestrierung kann für die oberste Viszeralarterie auch eine proximale Einkerbung (Scallop) von 10 oder 20 mm Breite geordert werden (Abb. 1). Die Endoprothese weist durchgehende ringförmige Z‑Stents aus Stahl (in der neuesten Generation auch aus Nitinol) auf; dementsprechend können die Fenestrierungen nicht völlig frei positioniert werden, wenn eine metallfreie Öffnung gewünscht wird, wie es für die Platzierung eines Verbindungsstentgrafts erforderlich ist. Insbesondere bei sehr nahe zusammenliegenden Ostien gibt es Einschränkungen dahingehend, dass unter Umständen ein Ostium von einem Stentdraht gekreuzt wird, wodurch dort kein Verbindungsstentgraft eingesetzt werden kann.

Der ZFEN bietet als einziges Fabrikat die Möglichkeit, eine Freisetzung mit zunächst 70 % des Durchmessers durchzuführen, wobei hier der Stentgraft an der posterioren Zirkumferenz gerafft ist (sog. „diameter-reducing ties“). Der Operateur sondiert üblicherweise von der Gegenseite über einen femoralen Zugang das untere Ende des partiell expandierten tubulären Stentgrafts und platziert über eine 20- bis 22-F-Schleuse bis zu 4 Drähte durch die jeweiligen Fenestrierungen in die zugehörige Viszeralarterie. Auch können kleinere Schleusen über diese Drähte vorgelegt werden. Während der Sondierung sind aufgrund der nicht vollständigen Expansion noch kleinere Korrekturen der Rotation und Höhe des Stentgrafts möglich. Erst nach Vorlegen der Drähte/Schleusen wird die Raffung aufgelöst und der Stentgraft expandiert zum vollen Durchmesser, wobei die Fenestrierungen exakt an die Ostien der jeweiligen Viszeralarterie heranführen. Danach werden sequenziell die Verbindungsstentgrafts gesetzt. Den Abschluss der Prozedur bildet üblicherweise das Setzen einer speziell für den ZFEN konstruierten dreiteiligen Bifurkationsprothese.

Mehrere Studien zeigen ausgezeichnete Erfolgsraten und eine niedrige Morbidität und Mortalität auch im Langzeitverlauf [20]. Die Ergebnisse von Implantationen mit 4 Fenestrierungen zeigen ähnlich gute Daten im Vergleich zu zweifach fenestrierten Implantationen, wobei ein Vorteil des vierfach fenestrierten Zugangs darin besteht, dass die suprazöliakale Stentkomponente im Bedarfsfall relativ einfach nach proximal verlängert werden kann, sollte die Krankheit im Intervall fortschreiten [12, 21].

### Fenestrierte Anaconda

**Figure 2:**
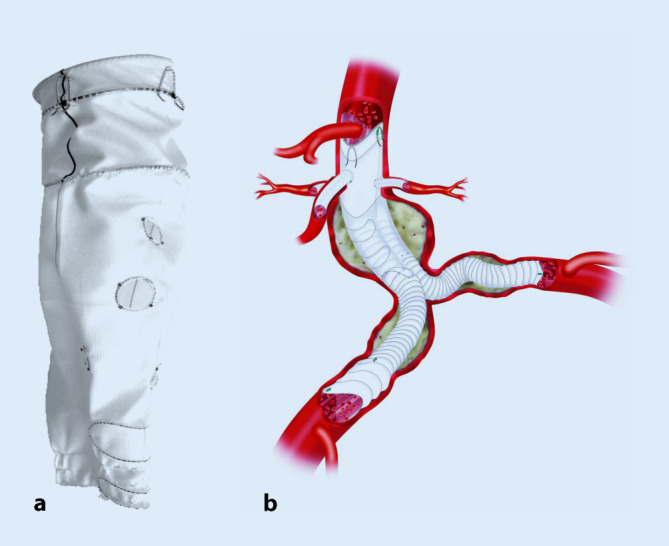

Die fenestrierte Anaconda (Terumo Aortic, Tokyo, Japan) basiert auf dem dreiteiligen infrarenalen Anaconda-System, wobei Fenestrierungen als patientenbezogene Sonderanfertigung sowohl in einem tubulären Hauptkörper als auch in einem Bifurkationsstentgraft oder auch einem iliakalen Bein angefertigt werden können. Der proximale Abschluss des Anaconda-Hauptkörpers besteht aus 3 Ringstents, die eine wellige, *fischmaulartige* Konfiguration aufweisen und die bis zur vollständigen Ablösung vom Einführsystem kollabiert und repositioniert werden können. Während bei der konventionellen infrarenalen Anaconda die *Täler* der Ringstents lateral an den Nierenarterien positioniert werden, erfolgt bei der zweifach fenestrierten Anaconda die Positionierung des Tals in der anterioren und posterioren Orientierung, um die A. mesenterica superior aufzunehmen (Abb. 2). Im Gegensatz zur Cook-Endoprothese weist die Anaconda distal der 3 Abdichtungsstents im Bereich der Fenestrierungen keine Metallstents auf, und das Graftmaterial legt sich alleine durch den Blutdruck an die Aortenwand an. Erst weiter distal finden sich wieder Ringstents. Dies hat den Vorteil, dass die Fenestrierungen völlig frei positioniert werden und auch knapp nebeneinander abgehende Viszeralarterien versorgt werden können. Ein Nachteil besteht darin, dass die Prothese speziell bei größeren Repositionierungen nach kaudal einfalten oder kollabieren kann. Vor dem Setzen der Verbindungsstentgrafts sollte auch auf eine optimale Apposition der Fenestrierung an der Aortenwand geachtet werden. Die Sondierung der Fenestrierungen erfolgt üblicherweise sequenziell von der kontralateralen Seite her, von kaudal nach kranial, was kleinere Repositionsmanöver möglich macht. Es besteht die Möglichkeit, im unverstärkten Stentkörper um die Fenestrierungen Drahtverstärkungen zu platzieren, um bei kritischen Angulationen einer Einfaltung vorzubeugen. Eine Besonderheit bei der fenestrierten Anaconda besteht darin, dass die Prothese stets zweifach produziert wird, wobei zunächst die Lieferung einer unsterilen Testprothese und eines anhand der CT-Daten 3‑D-gedruckten Aortenmodells erfolgt, an dem der Operateur eine Testimplantation vornimmt. Werden hier Schwierigkeiten an der Sondierbarkeit festgestellt, kann die endgültige sterile Prothese noch abgeändert werden. Für die Zukunft ist anstelle der physischen Testimplantation eine Computersimulation geplant, die dann auch die Elastizität der Aorta und die Wirkung des Blutstroms berücksichtigen kann.

Mehrere große Serien haben bei adäquater Komplikations- und Mortalitätsrate eine fehlende Progression des Aneurysmasacks in 83–99 % der Fälle beschrieben [5, 23].

### Verzweigte Endoprothesen

Im Gegensatz zu Fenestrierungen kommen Verzweigungen dort zur Anwendung, wo die Viszeralarterie bereits aus einem dilatierten Anteil der Aorta abgeht und eine Fenestrierung nicht mehr an der Aortenwand anliegen könnte [17]. Verzweigungen sind in der Regel gemäß dem Blutfluss von kranial nach kaudal orientiert, in Einzelfällen sind aber, etwa im Fall von steil nach kranial abgehenden Nierenarterien, auch kranial orientierte Verzweigungen möglich. Für außenliegende Verzweigungen ist ausreichend Platz erforderlich: So sollte das Aortenlumen den Hauptkörper von 16–20 mm und die 6–8 mm breite Aufzweigung ohne wesentliche Kompression aufnehmen können, was im Fall von Aneurysmen im Rahmen einer Dissektion unter Umständen eine Spaltung des Endothelschlauchs auf Höhe der Viszeralarterien erforderlich machen kann. Nach kaudal orientierte Verzweigungen werden mit der Spitze 10–20 mm oberhalb des Viszeralarterienostiums abgesetzt und wurden bisher von einem brachialen Zugang her von kranial sondiert. Diese Technik weist einige Nachteile auf: Der Zugangsweg ist lang, erstreckt sich über eine möglicherweise erkrankte thorakale Aorta und ist mit einem bis zu 4 %igen Risiko eines Insultgeschehens assoziiert [4]. Auch führt die Sondierung mitunter zu einer erheblichen Zunahme der Operationszeit und der Strahlendosis. Erfahrungen in unserem Zentrum zeigen, dass mit Hilfe der neueren steuerbaren Schleusen (z. B. Heli FX, Medtronic) eine Sondierung auch fast immer von einem rein femoralen Zugang erfolgen kann, was zu einer signifikanten Verkürzung der Sondierungszeit führt [9].

Im Gegensatz zu Fenestrierungen erlauben Verzweigungen einen größeren Spielraum bei der Sondierung des jeweiligen viszeralen Gefäßes, und eine vorgefertigte Konfiguration einer vierfach verzweigten Prothese ist mit geringen Einschränkungen für einen weiten Bereich unterschiedlicher Anatomien einsetzbar [3]. Dies ermöglicht erstmalig auch die endovaskuläre Versorgung von akuten und subakuten Patienten [11].

### Cook Medical BEVAR-Programm

**Figure 3:**
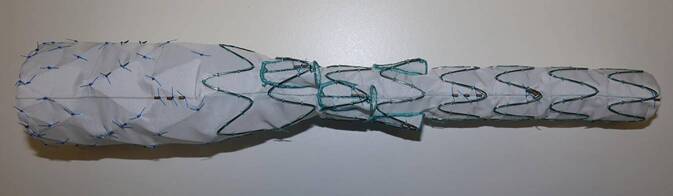

Der T‑branch von Cook Medical ist eine CE-zertifizierte Endoprothese mit einer Länge von 202 mm und 4 Verzweigungen bei 12 Uhr, 1 Uhr, 10 Uhr und 3 Uhr für die akute Versorgung von Viszeralarterien [3]. Der proximale Durchmesser beträgt 32 mm, der distale Durchmesser 18 mm, und zumindest distal muss in den meisten Fällen mit einer Bifurkationsprothese die Abdichtung in den Beckenarterien vorgenommen werden (Abb. 3). Je nach Aortendurchmesser am thorakoabdominellen Übergang kann die Prothese kranial primär abdichten oder mit einem tubulären thorakalen Stentgraft verlängert werden. Als patientenspezifische Anfertigung bietet Cook als einziger Hersteller auch Kombinationen aus Verzweigungen und Fenestrierungen an. Diese kommen beispielsweise dort zur Anwendung, wo einzelne Viszeralarterien aus dem Aneurysma und gleichzeitig andere aus einem engen Hals abgehen. Zur Erleichterung der Sondierung von inneren oder äußeren Verzweigungen werden (auf Kosten des Durchmessers des Einführbestecks) auch vorgelegte Katheter angeboten, da speziell bei der kranialen Sondierung von inneren Verzweigungen über Schwierigkeiten berichtet wurde. Diese vorgelegten Katheter werden allerdings bei der weiteren Verbreitung der Verwendung von steuerbaren Schleusen und der femoralen Sondierung, mit denen das Aufsuchen und Katheterisieren einer Aufzweigung (egal ob innere oder äußere) nur wenige Sekunden dauert, obsolet werden.

### E-nside- und E-xtra-Programm

**Figure 4:**
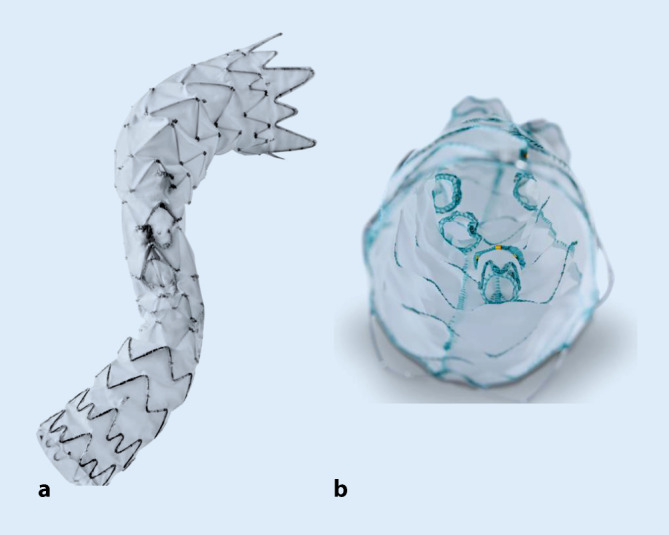

Die Jotec/Artivion GmbH (Hechingen, Deutschland) bietet neben individuell patientenbezogenen inneren und äußeren Verzweigungen auch eine vorgefertigte Endoprothese mit 4 inneren Verzweigungen an. Innere Verzweigungen können als Synthese von Fenestrierungen und äußeren Verzweigungen gesehen werden. Der innere Zweig bietet eine ca. 15 mm sichere Abdichtungszone für den Verbindungsstentgraft und öffnet sich distal trichterförmig, wodurch die Verbindungsstentgrafts in unterschiedlichen Winkeln abgehen können, ohne signifikante Impression oder Knickbildung. Der proximale Durchmesser der Prothese ist 33 mm oder 38 mm, was in einem größeren Prozentsatz der Fälle eine primäre kraniale Abdichtung erlaubt (Abb. 4). Eine anatomische Kohortenstudie zeigte die Anwendbarkeit der E‑nside-Prothese in 43 % eines Patientenkollektivs mit thorakoabdominellem Aortenaneurysma [2]. Patienten mit individuell angefertigten Jotec-Prothesen wiesen ähnliche Erfolgs- und Komplikationsraten auf wie vergleichbare Implantate [13]. Hinsichtlich des vorgefertigten E‑nside existieren noch keine größeren Studien, eine multizentrische Studien mit 13 europäischen Zentren und einem geplanten Kollektiv von 200 Patienten läuft seit Juli 2020 (ClinicalTrials.gov NCT 04383145).

### Verbindungsstentgrafts

Trotz der guten Ergebnisse der fenestrierten und verzweigten endovaskulären aortalen Reparatur liegen speziell im Bereich der VSG sowohl in der Forschung als auch in der Technologie noch Defizite vor. Speziell im Bereich des BEVAR wurden VSG-assoziierte Reinterventionsraten von 33 % nach 3 Jahren und bis zu 50 % nach 5 Jahren angegeben [6, 15, 21]. Obwohl seit Jahren die Stentgrafts von mehreren Herstellern für diesen Zweck verwendet werden, ist bis dato kein einziges Produkt für die Verwendung als Verbindungsstentgraft CE-zertifiziert. Nach der Erfahrung der Autoren sind speziell Brüche und Endoleaks in VSG nach BEVAR typischerweise schwer zu diagnostizieren und die notwendigen Reinterventionen mit signifikanter Morbidität und Mortalität verbunden. In einer retrospektiven Fall-Kontroll-Studie wurden von 125 Verzweigungen in 54 Patienten in einer multizentrischen Komplikationsdatenbank 23 Stentbrüche in 12 Patienten identifiziert [10]. Typischerweise traten die Brüche in einschichtigen Produkten auf, bei stärkeren Angulationen im Verbindungsstengraft und gehäuft in einzelnen Patienten (d. h. nach dem Bruch in einem VSG war das Auftreten eines Bruchs in einem anderen Stentgraft wahrscheinlicher).

Die Resultate legen Folgendes nahe:

1. Beim BEVAR sollten keine einschichtigen Verbindungsstentgrafts verwendet werden.
2. Bei der CT-Überwachung nach BEVAR sollte nach Möglichkeit mittels hochauflösender CT-Scans und zusätzlicher Rekonstruktion im Knochenkernel das Frühstadium (d. h. der Bruch einzelner Stentdrähte) identifiziert werden, bevor eine komplette Separation im VSG eingesetzt wird.
3. Speziell bei Patienten, die bereits einen Stentbruch hatten, ist eine engmaschige Überwachung durchzuführen.

Mehrere Hersteller bieten Stentgrafts in passenden Größen an, die als VSG für FEVAR/BEVAR verwendet werden: Beim Advanta V12 (Getinge) handelt es sich um einen ballonexpandierbaren Stahlstent, der auf beiden Seiten von einer extrudierten Polytetrafluorethylen(ePTFE)-Schicht umgeben ist. Der iCover(iVascular)-Stent besteht ähnlich dem Advanta aus einem beidseits mit PTFE umgebenen Stahlstent.

Im Gegensatz dazu weisen der BeGraft und BeGraft plus (Bentley InnoMed) einen Kobaltchromstent auf, der einseitig mit einer äußeren PTFE-Membran bedeckt ist. Die Membranstärke wurde 2015 von 0,1 mm auf 0,2 mm erhöht. 2018 wurde das BeGraft-plus-System eingeführt, welches aus zwei ineinandergelegten BeGraft-Stents besteht.

Jotec vermarktet unter dem Namen Eventus das baugleiche einschichtige BeGraft-System. Der Viabahn-VBX-Stentgraft (W.L. Gore) besteht aus einem Stahlstent, beidseitig umgeben von PTFE, wobei im Gegensatz zu den anderen Produkten die Stentringe untereinander keine Metallbrücken aufweisen, sondern ausschließlich durch die Membran verbunden sind.

Neben diesen ballonexpandierbaren Stents stellen Gore und Bard auch selbstexpandierbare Stents her. Rezente Analysen zeigen keinen signifikanten Unterschied zwischen selbstexpandierenden und ballonexpandierenden Stents in Bezug auf Undichtigkeiten im Sinne von Typ-3-Endoleaks [19].

## Fazit für die Praxis

- Fenestrierte und verzweigte Aortenprothesen erlauben die sichere Versorgung komplexer abdomineller Aortenpathologien wenn diese die Viszeralarterien miteinschließen.
- Zwar sind die meisten dieser Endoprothesen individuelle Sonderanfertigungen mit entsprechender Lieferzeit, zwei vierfach verzweigte Endoprothesen in Standardausführung ermöglichen jedoch auch die Behandlung akuter Fälle.
- Verbindungsstentgrafts, die den Hauptkörper der Endoprothese mit den Viszeralarterien verbinden, stammen durchgehend von Fremdherstellern, sind nicht für diese spezielle Anwendung zertifiziert und stellen oft einen Grund für Reinterventionen dar.
- In der Nachsorge muss diesen Verbindungsstents besondere Aufmerksamkeit im Hinblick auf Knickbildungen und Brüche geschenkt werden.
- Von der Verwendung einschichtiger Verbindungsstents sollte beim BEVAR („branched endovascular aneurysm repair“) Abstand genommen werden.
